# Comparative analysis of translatomics and transcriptomics in the longissimus dorsi muscle of Luchuan and Duroc pigs

**DOI:** 10.1371/journal.pone.0319399

**Published:** 2025-03-18

**Authors:** Songtao Su, Hailong Hu, Kang Liu, Siqi Liu, Zupeng Luo, Jingsu Yu, Tianyu Jiang, Xiangling Li, Chang Sun, Lin Yu, Yuehui Liang, Lei Zhou

**Affiliations:** Institute of Digestive Disease, Guangxi Academy of Medical Sciences, the People’s Hospital of Guangxi Zhuang Autonomous Region, Nanning, China; South China Agricultural University, CHINA

## Abstract

IMF (Intramuscular fat) content is a crucial indicator of meat quality in the livestock industry. However, the molecular mechanisms underlying IMF deposition remain unclear in pigs. In this study, we conducted RNC-seq (ribosome nascent-chain complex-bound RNA sequencing) and RNA-seq (RNA sequencing) analyses on the longissimus dorsi muscle of Duroc pigs (a lean breed) and Luchuan pigs (a fat breed) to uncover the genetic basis for the divergent IMF content. The results show that the overall translation level of Luchuan pigs is significantly higher than Duroc pigs, while there is no significant difference in the transcription level. Enzymes related to fatty acid synthesis and elongation, such as ACACA, FASN, and ELOVL5, are significantly up-regulated at the translation level, while enzymes associated with fatty acid degradation, namely ALDH1B1 and ALDH2, are significantly down-regulated. However, there is no significant difference in their transcription levels. qRT-PCR and Western Blotting experiments for ELOVL5 confirm the reliability of the sequencing results. Additionally, the translation initiation factor eIF4A1, known to positively regulate gene translation, displayed higher expression in Luchuan pigs rather than in Duroc pigs and the 5’UTR structural features of genes involved in translation up-regulation matched the mRNA selectivity of eIF4A1. In conclusion, these findings suggest the up-regulation of the eIF4A1 gene expression in Luchuan pigs may elevate the translation levels of genes related to lipid synthesis through translational regulation, further resulting in an increase in IMF content.

## 1. Introduction

IMF, known as marbling in the meat industry, greatly influences the marketing of fresh meat, especially beef and pork loin cuts [1]. Therefore, exploring the mechanisms of IMF deposition holds great importance in animal husbandry. LM (Luchuan pig) is one of the famous native breeds in China, known for its high IMF, while DM (Duroc pig) is one of the main commercial pig breeds, characterized by its low IMF content. However, the genetic basis for the differential IMF content between different pig breeds is still unclear. Therefore, DMs and LMs have become ideal models for studying the molecular mechanisms of IMF deposition.

More studies have shown that protein abundance correlates with mRNA abundance at a low level, but one study showed that the correlation coefficient with RNC-mRNA abundance exceeded 0.9 [2–5]. A quantitative model has demonstrated that protein abundance is predominantly controlled at the translational level, accounting for more than half of all regulatory amplitudes [6]. The translation process can be divided into initiation, elongation, termination, and ribosomal cycle, with most regulation occurring in the initiation phase, which determines the proportion of translated mRNAs which are the mRNAs bound to the ribosome-nascent chain complex (RNC-mRNA) [7]. The translation initiation process in eukaryotes is more complex, and eukaryotic initiation factor (eIF) 4A, a DEAD-box RNA helicase, plays a key role. Mammalian cells encode two eIF4A paralogs, eIF4A1 and eIF4A2. Unlike eIF4A2, eIF4A1 is generally a more abundant protein and is essential for cellular viability [8]. Moreover, recent studies have shown that eIF4A1 is dependent on the mRNA 5’UTR and prefers to bind mRNAs with GC-rich, long, and structurally complex 5’UTRs [9,10].

By combining RNC-seq with RNA-seq, it is possible to gain a more comprehensive understanding of the relationship between mRNA abundance and translation efficiency [11]. Furthermore, the mRNA translation ratio (TR, defined as the abundance ratio of RNC-mRNA to the total mRNA of the gene) can respond to the translation initiation efficiency of the gene [5,12,13]. However, the studies using RNC-seq and RNA-seq to detect the gene translatome and transcriptome levels in the longissimus dorsi muscle tissue of DMs and LMs has not been reported.

In this study, through the combined analysis of translatomics and transcriptomics, we identified key genes that differ between the two pig breeds in terms of IMF and revealed the molecular mechanisms underlying IMF deposition, which provide references for enhancing IMF content.

## 2. Materials and methods

### 2.1. Animals and samples collection

Three DMs and three LMs were used in this study and all were fed normally under the same conditions (Table 1) [14]. DMs and LMs were purchased from Guangxi State Farms Yongxin Animal Husbandry Group Co. Ltd. and The Animal Husbandry Research Institute of Guangxi Zhuang Autonomous Region, respectively. All pigs were euthanized at 180 days of age and fasted overnight before execution. Samples of the longissimus dorsi muscle from each pig were collected. Euthanasia was performed according to the Chinese industry standard (GBT 17236–1998) by electric stunning followed by exsanguination. All animal experiments have been approved by the Animal Ethics Committee of Guangxi University (GXU2015-003).

**Table 1:** The dietary nutritional levels used in this study.

Dietary nutritional level	Content (%)
Energy (KJ/kg)	11.98
Crude protein (%)	16
Crude fiber (%)	6
Crude ash (%)	7
Calcium (%)	0.6-1.2
Total phosphorus (%)	0.4-1.0
NaCl (%)	0.2-0.8
Lysine (%)	0.8

### 2.2. TG (triglyceride) measurement

The TG content of the longissimus dorsi muscle samples was determined using an assay kit (Nanjing Jiancheng, China) as described previously [15,16]. Protein concentrations were determined by the BCA protein quantification kit (P0009; Beyotime Institute of Biotechnology, China). The TG level was normalized to the protein concentration.

### 2.3. RNC-seq and RNA-seq

RNC isolation was performed as previously described [17], 90 mg of tissue was treated with 200 μL lysis buffer (1% Triton X-100 [GBCBIO Technologies Inc., Guangzhou, Guangdong, China] in ribosome buffer [20 mM HEPES, 2 mM dithiothreitol, 15 mM MgCl2, 200 mM KCl and 100 μg/mL cycloheximide]) and then homogenized. An additional 700 μL of lysis buffer was added to the samples, followed by incubation in an ice-bath for 30 min. After centrifuging at 16,200 x g for 20 min at 4°C, the supernatants were carefully added to the surface of 5 mL of sucrose buffer (30% sucrose in ribosome buffer), and the RNC complex was pelleted by ultra-centrifugation at 330,000 x g for 3 hours at 4°C. RNC-RNA was extracted from the RNC complex using TRIzol. The quality of the total RNA and RNC was detected by electrophoresis using a 2.5% agarose gel. Three samples from 3 independent experiments were used for subsequent RNA-seq to assess mRNA and RNC-RNA.

#### 2.4. qRT-PCR (Quantitative real-time polymerase chain reaction)

The RNA-seq results were validated using qPCR. Total RNA was extracted using the TRIzol reagent (Invitrogen, USA) and cDNA was synthesized by reverse transcription with M-MLV enzyme (Takara, Dalian, China) and random primer oligo-dT18, according to the manufacturer’s instructions. qRT-PCR was conducted on a qTOWER3G PCR system (Analytikjena) using 2x Realstar Green Fast Mixture (Takara Bio Inc., Japan) with β-actin as the internal reference gene. The reaction system was: 95°C for 15 seconds, 60°C for 1 minute, 72°C for 10 seconds, cycled 40 times, followed by 72°C for 5 minutes, and 12°C for 2 minutes. The relative gene expression was determined using the 2^-ΔΔCt^ method. Primer design and specificity testing were performed using the Primer–BLAST online tool from NCBI (https://www.ncbi.nlm.nih.gov/tools/primer-blast), and the primers were synthesized by Beijing Qingke Xinye Biotechnology Co., Ltd. The primers were as follows:

ELOVL5: Forward primers - ACGCAGTGGAGGAGAA, Reverse primers - TACAGCCAGCCGAGA
eIF4A1: Forward primers - AGGATCATGTCTGCGAGTCAG, Reverse primers - TTGGGCTTGAGCGATCACAT.

### 2.5. Sequencing analysis

Both the RNA-seq and RNC-seq datasets were mapped to the *RefSeq mRNA reference sequence* (Sus scrofa.Sscrofa11.1) using the *FANse3 algorithm* with the following parameters: –L80 –E5 –I0 –S14 –B1 –U0 [18,19]. Reads that mapped to alternative splice variants of one gene were merged. The mRNA and RNC-mRNA in each sample were normalized using *rpkM* [20], while the relative abundance between two groups was normalized using the *edgeR* package [21]. Differentially expressed mRNAs and RNC-mRNAs were identified using the *edgeR* package with a *fold-change* (FC) cutoff of ≥  2 and a *p*-value (*P*) cutoff of <  0.05. The Benjamini-Hochberg correction was applied in multiple comparisons, with FDR <  0.05 considered significant. The TR of a gene of one sample was calculated as previously described: the quotient of the RNC-mRNA rpkM and the mRNA rpkM [5]. Differentially regulated TRs were calculated using a *t-test* with a | FC | >  1.3 and *P* <  0.05. Used the Gene Ontology official website (http://geneontology.org/) for GO enrichment analysis. Input the gene list and selected Sus scrofa as the reference background for the enrichment analysis. The enrichment included two parts: Biological Process (BP) and Cellular Component (CC). Used a *P* <  0.05 as the threshold for significant enrichment. Performed KEGG enrichment analysis using the official KEGG website (https://www.kegg.jp/), input the gene list, and chose ssc as the reference background for pathway enrichment. For the pathway map, used color to search for paths, with upregulated genes marked in red and downregulated genes marked in blue. Used Fisher’s *P* <  0.05 as the threshold for significant enrichment. The network diagram used the ClueGO plugin of Cytoscape for enrichment analysis [22]. Using the UniProt protein database (https://www.uniprot.org/), aligned the genes to the UniProtDB database to obtain the protein sequences, subcellular localization, biological functions, and pathways involved. Used the UCSC database (https://genome.ucsc.edu/) to obtain gene sequences. Aligned the gene list to the database and selected the 5’UTR or CDS sequence to download the Fasta file. RNA folding free energy was calculated using *linearFold* [23] with default parameters, using a thermodynamic-based model. RNA G-quadruplex propensity was calculated using the *pqsfinder* package [24]. Used the MEME website (https://meme-suite.org/meme/tools/meme), imported the target gene fasta file, selected the default parameters for motif searching, and exported the motif with the smallest *E*-value (the smaller the *E*-value, the higher the reliability).

### 2.6. Western blotting

The protein supernatant concentration was determined by the bicinchoninic acid method (BCA) (P0009; Beyotime Institute of Biotechnology, China). The sample was added to Protein loading buffer (Solarbio, Beijing, China) and boiled for 10 min at 100°C. The electrophoresis program was set to 75 V for 30 minutes, followed by 120 V for 55 minutes (Bio-Rad, California, USA). After electrophoresis, the transfer membrane program was set to 25 V for 20 minutes (Bio-Rad, California, USA). Anti-ELOVL5 (1:2000, Sangon Biotech, Shanghai, China) and anti-GAPDH (1:50000, proteintech, Wuhan, China) are primary antibodies. The primary antibodies were used according to the instructions in the antibody manual. The PVDF membrane (MILLIPORE, USA) was treated with HRP conjugated secondary antibody (1:3000) and ECL hypersensitive luminous liquid (GenStar, Beijing, China) and finally imaging was performed using the Bio-Rad Imaging System (Bio-rad, California, USA). Quantitative assessment of the bands was conducted using Image J software.

### 2.7. Statistical analysis

Mean ±  standard error was used to present the experimental results, while the *t-test* and Wilcoxonon rank sum test were employed to determine the significance of differences between two groups. Results with P-values less than 0.05 were deemed significant, and denoted with asterisks ( * for *P* <  0.05, ** for *P* <  0.01, and *** for *P* < 0.001).

## 3. Results

### 3.1. Overview of sequencing data of transcriptome and translatome

**Fig 1:**
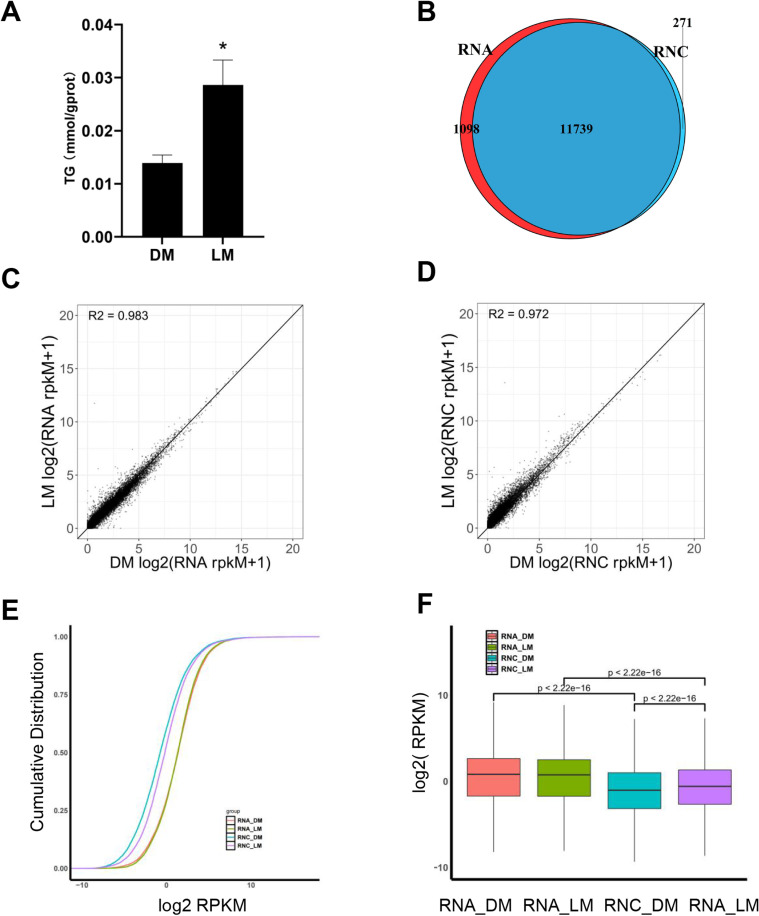
Overview of sequencing data of transcriptome and translatome. (A) TG concentrations in the longissimus dorsi muscle of two pig breeds (n = 3). (B) Venn diagrams of genes identified by RNA-seq and RNC-seq in LM group and DM group. (C, D) The linear relationship between DM and LM in mRNA abundance (rpkM) and RNC-mRNA abundance (rpkM). (E) Cumulative distribution curve of mRNA and RNC-mRNA abundance in LM group and DM group. The x-axis represents the logarithmic value of gene expression, and the y-axis represents the cumulative distribution. (F) Significance test for differences in gene expression among groups.

The significantly higher TG content in the longissimus dorsi muscle of LMs compared to DMs indicates a higher IMF content in LMs (*P* <  0.05) (Fig 1A). To elucidate the causes of the aforementioned phenomenon, we conducted RNA-seq and RNC-seq on the longissimus dorsi muscles of these two types of pigs, identifying approximately 12,800 and 12,000 genes, respectively (S1 Table in S1 File, Fig 1B). Comparing the genes identified by RNA-seq and RNC-seq, 11739 genes were found to overlap, 1098 genes were identified only by RNA-seq, and 271 genes were identified only by RNC-seq (Fig 1B), indicating most of the mRNAs entered the translation process. The gene expression correlation heatmap showed that the RNA-seq and RNC-seq sequencing results had a high correlation between biological replicates (0.81 <  r <  0.99), indicating good quality of biological replicates. The correlation between mRNA and RNC-mRNA was slightly lower (0.7 <  r <  0.99), suggesting that there was a relationship between mRNA and RNC-mRNA expression levels, but also some differences (S1A Fig in S1 File). The gene expression distribution plot showed that the density curves of different sequencing methods for replicate samples overlapped significantly, further indicating good biological reproducibility of the samples. When comparing mRNA and RNC-mRNA, it could be observed that the peak of RNC-mRNA had shifted slightly to the left compared to that of mRNA, while their overall peak widths (representing the degree of dispersion or variance) were similar. This suggested that RNC-mRNA expression levels were generally lower than those of mRNA (S1B Fig in S1 File).

Additionally, the results of the regression analysis demonstrated a highly significant linear correlation between mRNA and RNC-mRNA levels in both pig breeds, with R2 values exceeding 0.9 (Fig 1C-D) while the linear correlation between mRNA and RNC-mRNA levels was slightly less pronounced, with R^2^ values above 0.8 (S1C-D Fig in S1 File), indicating a discrepancy between gene transcription and translation levels. Principal component analysis showed that the expression patterns of transcriptional level of genes in LM and DM pigs were similar, while the translation levels had big differences (S1E Fig in S1 File). Subsequently, we analyzed the cumulative distribution curves of gene expression in each group and observed the mRNA expression curves of both pig breeds nearly overlapped while the RNC-mRNA expression curves showed a leftward shift compared to mRNA. The RNC-mRNA expression curves of LMs exhibited a rightward shift in contrast to DMs (Fig 1E). Wilcoxon rank sum test showed the RNC-mRNA expression level was significantly lower than the mRNA expression level in both pig breeds, the RNC-mRNA expression level was significantly higher in LMs than in DMs, but the mRNA expression level was not significantly different (Fig 1F).

### 3.2. Differentially expressed genes in transcriptome and translatome

**Fig 2:**
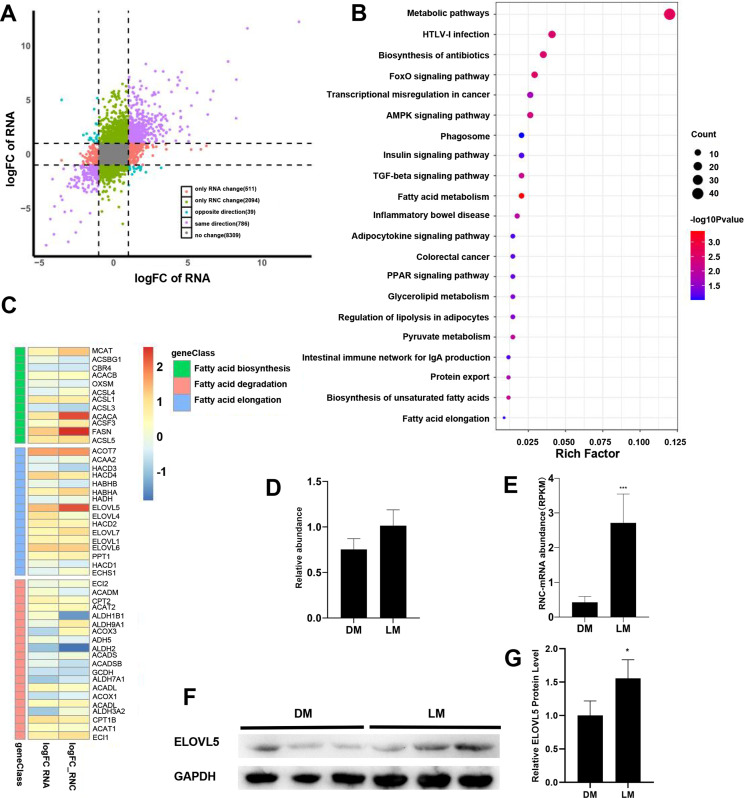
Differentially expressed genes analysis in transcriptome and translatome. (A) Nine-quadrant plot of mRNA vs. RNC-mRNA fold-change. (B) Significant enriched KEGG pathways by RNC-only. (C) Heatmap of genes related to fatty acid metabolism. (D) QRT-PCR to verify the relative mRNA expression of ELOVL5. (E) ELOVL5 RNC-mRNA abundance measured by RNC-seq. (F) Western-bolt to verify the protein abundance of ELOVL5. (G) Quantitative analysis of ELOVL5 bands using ImageJ software. (RNA-only: Genes that are significantly differentially expressed only at the transcriptional level; RNC-only: Genes that are significantly differentially expressed only at the translation level.).

Differentially expressed mRNAs (DEmRNAs) and Differentially expressed RNC-mRNAs (DERNCs) were identified using the R package edgeR, based on | FC | ≥  1 and *P* <  0.05, using DMs as control group [20]. In total, 232 up-regulated and 425 down-regulated mRNAs, and 281 up-regulated and 419 down-regulated RNC-mRNAs were identified (S2A-B Fig in S1 File). DEmRNAs were quantitatively similar to DERNCs, and comparison of the two sets of differentially expressed genes revealed only 199 genes overlapped (S2C Fig in S1 File). To further analyze the differences in gene expression in the translatome and transcriptome of the two pig breeds, the genes were classified into five categories according to their FC: same change, reverse change, RNC-mRNA change only, mRNA change only, and no change. Through the above-mentioned methods, 2094 genes with RNC-mRNA change only and 511 genes with mRNA change only were identified, further indicating the differences in gene translation levels were more significant than those in transcription levels (Fig 2A).

KEGG pathway enrichment suggests genes with only RNC-mRNA changes are involved in many pathways related to lipid metabolism, such as fatty acid elongation, fatty acid synthesis, and PPARγ signaling pathway (Fig 2B). We selected the pathways related to lipid metabolism for demonstration, The results showed that the translation levels of genes related to lipid synthesis were significantly higher in LMs than in DMs, while most of the genes within the FoxO signaling pathway related to myoblast proliferation were down-regulated (S2D Fig in S1 File). We counted the number and types of enzymes encoded by different expression genes and found that many of the enzymes encoded by genes with RNC-mRNA changes only were involved in lipid metabolic processes and protein modification (Table 2), while more enzymes were encoded by genes with RNC-mRNA changes only than by genes with mRNA changes only (S2E Fig in S1 File). The clustering heat map shows the key enzymes for fatty acid synthesis and elongation, ACACA, FASN, and ELOVL5, are significantly up-regulated at the translation level, while ALDH1B1 and ALDH2 for fatty acid degradation are significantly down-regulated, but their transcript levels are not significantly different (Fig 2C). We quantified the expression of ELOVL5 (extra long chain fatty acid elongase 5) in the longissimus dorsi muscle of the two pig breeds to verify the reliability of the sequencing data. The qRT-PCR results showed the expression of ELOVL5 in LMs was not significantly different from DMs (*P* >  0.05) (Fig 2D). But RNC-seq data showed the translation level of ELOVL5 was significantly higher in LMs than in DMs (Fig 2E). To verify whether there is a difference in the translation level of ELOVL5, we used western-blot to verify the protein expression level of ELOVL5, and the results showed the protein expression level of ELOVL5 was significantly higher in LMs than in DMs (Fig 2F-G).

**Table 2:** Number of the corresponding proteins of different expression genes involved in metabolic pathways.

Count Pathway	Only mRNA change	Only RNC-mRNA change
Lipid metabolism	7	13
Protein Modification	14	21
Glucose metabolism	5	2
Amino acid metabolism	5	7

### 3.3. Analysis of the TR between two pig breeds and functional enrichment of differential TR genes

**Fig 3:**
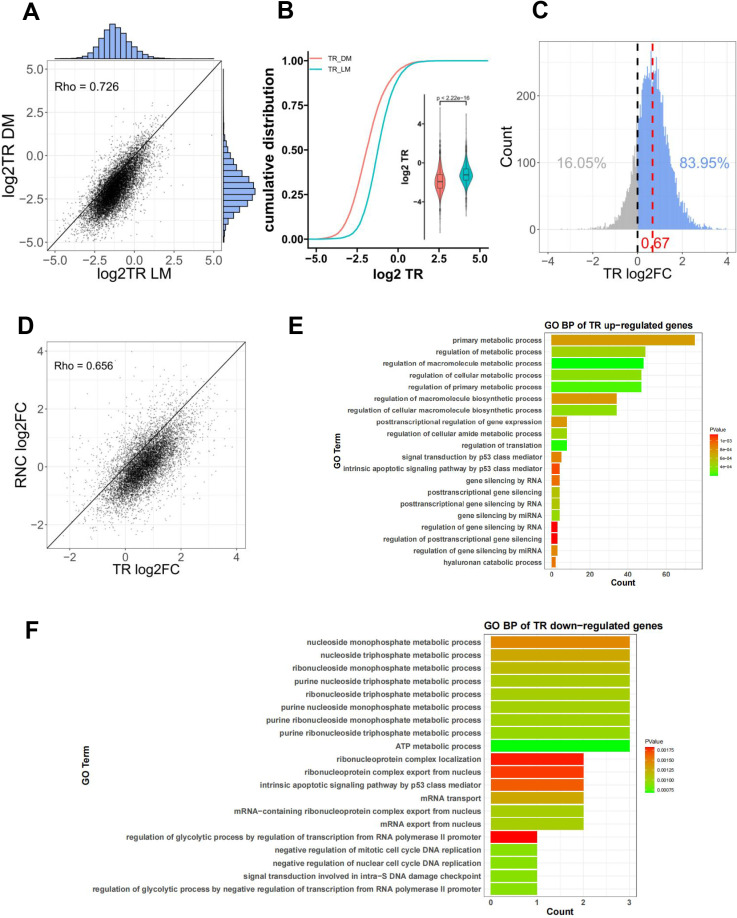
Analysis of the TR between two pig breeds and functional enrichment of differential TR genes. (A) Scatter plot of TR for two pig breeds. (B) The Wilcoxonon rank-sum test for TR differences between two pig breeds. (C) The distribution of TR log2FC. (D) The relationship between TR log2FC and RNC-mRNA log2FC. (E, F) Enrichment of GO biological processes for up-regulated TRs (E) and down-regulated TRs (F).

RNC-mRNA abundance is considered to be the basal translation level of the gene. TR can respond to the translation initiation efficiency of the gene [5,12,13]. From the scatter plot, it can be seen the TR between two pig breeds has a high correlation (Spearman’s rho =  0.726), and the points are mostly clustered below the diagonal, indicating the TR of LM is higher than DM (Fig 3A). The test results confirm this difference (*P* < 2.2e-16) (Fig 3B). DMs were used as the control group and the FC of TRs was calculated for the two pig breeds. We found there were more genes with FC greater than 0 (83.95%) than those less than 0 (16.05%), and an average log2FC is 0.68 (Fig 3C), which indicating the translation initiation efficiency of most genes in LMs increased relative to DMs. We also found FC of translation initiation efficiency was positively correlated with FC of basal translation level (Rho =  0.656) (Fig 3D), but very low correlation with FC of transcription level (Rho =  -0.141) (S3A Fig in S1 File). These results indicate that the gene translation initiation efficiency of LMs is higher than that of DM.

To further understand the function of the differential TR gene, subsequent analyses were conducted. The ratio of TR values between DMs and LMs was calculated as the differential FC, and a t-test was performed. A threshold of | FC | >  2 and *P* <  0.05 was employed, with DMs used as the control, and 20 up-regulated and 136 down-regulated TR genes were identified (S3B Fig in S1 File), enrichment of GO biological processes for differential TR genes revealed that this fraction of genes was significantly enriched in metabolism-related processes and the P53 signaling pathway (Fig 3E-F). Functional enrichment analysis of genes with very small TR differences (|FC|<0.5) revealed that these genes were mainly enriched in the basic life processes of the organism, such as animal organ morphogenesis, gland development etc. (S3C Fig in S1 File).

### 3.4. Analysis of translation initiation factors and their relationship with the characteristics of differential TR genes

**Fig 4:**
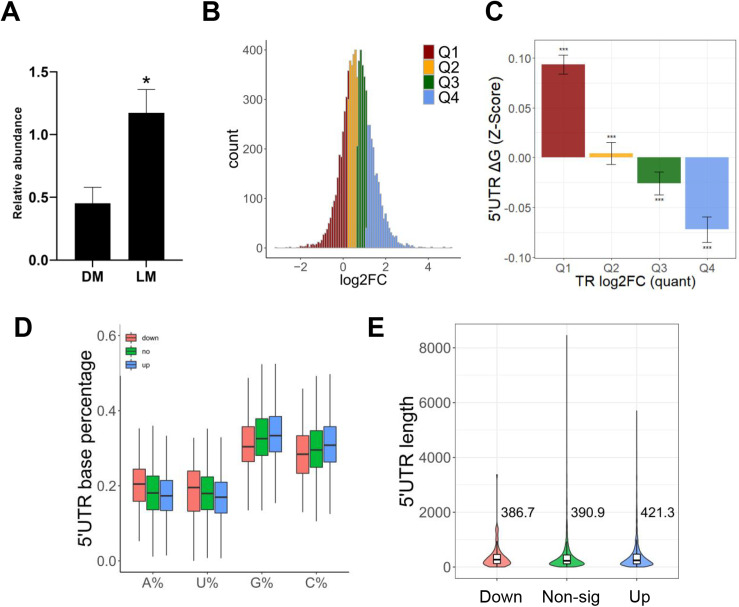
Analysis of translation initiation factors and their relationship with the characteristics of differential TR genes. (A) Relative expression of eIF4A1 in DM and LM. (B) The distribution of TR log2FC and the location of the quartiles are shown. (C) Folding free energy of the 5’UTR of genes with different quartiles of TR log2FC. (D) 5’UTR base ratios of differential TR genes. (E) Comparison of the 5’UTR lengths of differential TR genes.

To identify possible mechanisms for differences in the translatome, we analyzed the expression of all translation initiation factors in the longissimus dorsi muscle of the two pig breeds and found only eukaryotic translation initiation factor eIF4A1 was significantly differentially expressed (S2 Table in S1 File). eIF4A1, a DEAD-box RNA helicase, plays a key role in translation initiation. It has ATP-dependent helicase activity and is a key factor that affects the translation initiation efficiency [25]. The qRT-PCR results revealed a significantly higher expression level of eIF4A1 in LMs compared to DMs (*P* <  0.05) (Fig 4A).

To assess whether differences in TR are associated with the gene 5’UTR, we analyzed the structural features of the gene. First, we calculated the minimum free energies (ΔG) of the folded RNA secondary structure of the 5’UTR of RNA. mRNAs with more TR up-regulation (fourth quartile) have lower 5’UTR folding free energy (5’UTR is more stable) and mRNAs with more TR downregulation (first quartile) have higher 5’UTR folding free energy (Fig 4C) (Fig 4B shows the quantile information, the genes in Q1 are almost TR down-regulated genes). Then, we analyzed the 5’UTR lengths of genes and their base ratios. We found the 5’UTR of up-regulated TR genes had significantly lower A and U content compared to down-regulated TR genes, while the opposite was observed for C and G content (Fig 4D). Although there was no significant difference in 5’UTR length, there was a noticeable trend (Fig 4E).

### 3.5. Analysis of the characteristics of the 5’UTR in differentially expressed RNC-mRNA and their relationship with eIF4A1.

**Fig 5:**
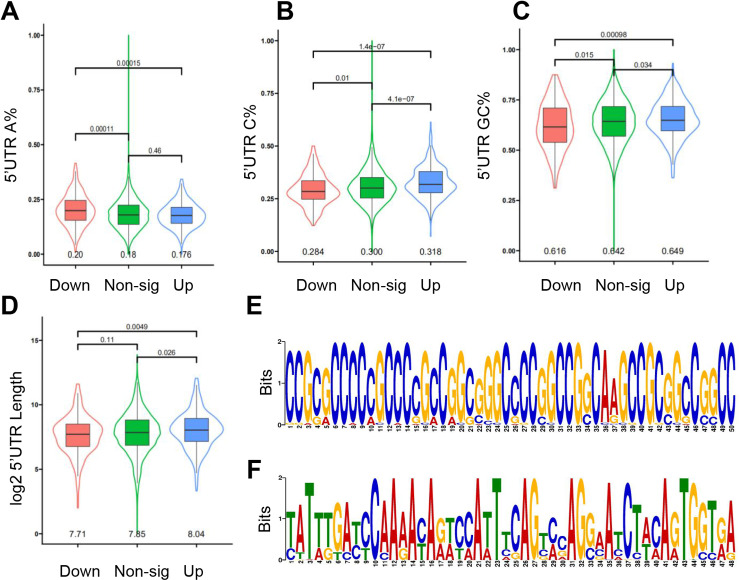
Analysis of the characteristics of the 5’UTR in differentially expressed RNC-mRNA and their relationship with eIF4A1. (A-C) Differences in the A, C, GC content of the 5’UTR of differentially expressed genes. (D) Differences in the length of the 5’UTR of differentially expressed genes. (E, F) Motif of the 5’UTR of the up-regulated genes (E) and the down-regulated genes (F). Select the motif with the smallest E value, which is the motif with the highest significance.

We analyzed the characteristics of differentially expressed RNC-mRNA. The results showed the A content of the 5’UTR of the up-regulated RNC-mRNA was significantly lower than the down-regulated gene (*P* <  0.05) (Fig 5A), the U and G content were both not significantly different (*P* >  0.05) (S3D-E Fig in S1 File), the C and GC content were both significantly higher than the down-regulated gene (*P* <  0.05) (Fig 5B-C). The length of 5’UTR of the up-regulated gene was significantly greater than the down-regulated gene (*P* <  0.05) (Fig 5D). We want to investigate whether the differences in translation levels between two pig breeds are associated with the preference of eIF4A1 for a high GC content in mRNA 5’UTR. In order to avoid the impact of transcriptional regulation on the expression levels of RNC-mRNA, we chose those genes where mRNA expression levels showed no significant differences but RNC-mRNA expression levels exhibited significant differences for analysis. Next, we used MEME with default parameters to identify motifs in the 5’UTR of those genes [26]. The analysis revealed a significant enrichment, with up-regulated genes displaying motifs enriched in G and C, whereas down-regulated genes exhibited enrichment in A and T (Fig 5E-F).

## 4. Discussion

In pig farming, IMF can affect the meat quality and economic value of pork [27]. IMF deposition is regulated by various genes and pathways. This study found the overall translation level of LMs is significantly higher than DMs, while there is no significant difference in transcription level (Fig 1E-F). The analysis of differentially expressed genes in transcriptome and translatome further indicating the differences in gene translation levels were more significant than those in transcription levels (Fig 2A). The majority of genes involved in the FoxO signaling pathway were downregulated, while most of the genes directly involved in lipid synthesis and those that indirectly positively regulated lipid metabolism were upregulated (S2D Fig in S1 File). FoxO was beneficial for maintaining the stemness of muscle stem cells, and its absence inhibited muscle growth [28]. Additionally, FoxO responded to fluctuating nutrients and regulated the metabolic homeostasis of various tissues and organs. FoxO also played a crucial role in lipid homeostasis by regulating lipogenesis, fatty acid oxidation, lipid transport, and cholesterol metabolism [29–31]. This was consistent with the higher intramuscular fat content and slower muscle growth rate observed in the Luchuan pig. At the same time, these genes showed no significant difference at the mRNA level, indicating that translational regulation played an important role in the phenotypic differences. Enzymes related to fatty acid synthesis and degradation show differences at the translation level but not at the transcription level (Fig 2C). FASN, ACACA, ACC1, and ELOVL5 play crucial roles in fatty acid synthesis, with FASN responsible for fatty acid synthesis, ACACA initiating long-chain fatty acid synthesis, ACC1 contributing to malonyl coenzyme A biosynthesis, and ELOVL5 involved in the elongation of long-chain fatty acids [32,33]. The abundance of these enzymes may influence the fatty acid metabolism process, thereby resulting in differences in IMF content between the two pig breeds. The qPCR and Western Blotting results for ELOVL5 confirm the reliability of the sequencing data (Fig 2D-G). These imply the main reason for the difference in IMF content may be translational regulation.

In general, the translation initiation process is a selective and highly regulated process [34,35]. Under normal physiological conditions, the regulation of protein synthesis at the level of translation initiation is essential for cell proliferation [36]. The analysis of TR between two pig breeds indicate the gene translation initiation efficiency of LMs is higher than DMs (Fig 3A-B). Enrichment of GO biological processes revealed differential TR genes were significantly enriched in metabolism-related processes and the P53 signaling pathway (Fig 3E-F). The main role of eIF4A1 is to decapacitate highly secondary structured RNA through its decapping enzyme activity and recruit the 43S pre-initiation complex (PIC) for successful assembly into mRNA, the rate-limiting step of translation initiation [37]. Only eIF4A1 was significantly differentially expressed in two pig breeds (S2 Table in S1 File). The expression of eIF4A1 in the longissimus dorsi muscle was significantly higher in LMs than in DMs (Fig 4A). Therefore, we hypothesized the high expression of eIF4A1 was responsible for the higher gene translation level in LMs than in DMs. Recent studies have shown eIF4A1 is selective for the structure of the mRNA 5’UTR, and it prefers to bind mRNAs with long, GC-rich, structurally complex 5’UTRs [9,10]. And the up-regulated TR genes may be associated with preferential binding of the translation initiation factor eIF4A1 to their 5’UTRs enriched in GC content (Fig 4D). In addition, the 5’UTR characteristics of differentially expressed RNC-mRNA coincide with the selectivity of eIF4A1 for mRNA (Fig 5D-F). Previous studies showed that eIF4A1 was crucial for B cell development and germinal center responses. After in vitro activation of B cells, eIF4A1 promoted the rate of protein synthesis [38]. eIF4A1-dependent mRNAs activated the local helicase activity of eIF4A1 through purine-rich 5’UTR sequences, which in turn promoted translation by facilitating the polymerization of eIF4A1. The AG motif in the 5’UTR of eIF4A1-dependent mRNAs specifically activated eIF4A1, promoting the assembly of eIF4A1 polymer complexes with helicase activity. These complexes were then localized near stable local RNA structures, allowing ribosomal subunit scanning and thereby enhancing the translation of eIF4A1-dependent mRNAs in the cell [39]. eIF4A limited intermolecular RNA-RNA interactions in cells, establishing eIF4A and other potential DEAD-box proteins as ATP-dependent RNA chaperones with an important role in restricting RNA aggregation, similar to the function of proteins like HSP70 in combating protein aggregates [40]. In addition, in Wujin pigs, the high-protein diet mainly promoted muscle protein accumulation by upregulating the expression of eIF2B1, eIF4B, and eIF4E during growth stages, rather than eIF4A1 [41]. Currently, there are few research reports on eIF4A1 in the field of animal husbandry, and our study is consistent with the previous studies mentioned above. These results are sufficient to suggest the up-regulation of translation levels in LMs is caused by the translational regulatory effect of eIF4A1.

In conclusion, our study suggests the up-regulation of eIF4A1 expression levels in the longest dorsal muscle of LM pigs may be responsible for the elevated translation levels in LM pig, particularly leading to the up-regulation of translation levels in lipid synthesis-related genes. These further promote increased lipid synthesis in LM pig muscles, accumulation of TG, and ultimately results in a higher IMF content in LM pigs compared to DMs (S3F Fig in S1 File). Our work definitely provides new insights for elucidating the molecular basis for IMF formation.

## Supporting information

S1 File **S1 Fig.** (A) HeatMap of gene expression of each group. Using Pearson correlation coefficient, where red indicates r is close to -1 and blue indicates r is close to 1. (B) Gene expression density curve for each group. Log-transformation of RNA-seq and RNC-seq gene expression levels (rpkM) for each sample to reduce their dispersion, followed by the creation of density plots for each. (C, D) The linear relationship between mRNA abundance and RNC-mRNA abundance. (E) Principal Component Analysis (PCA) result. **S2 Fig.** (A, B) Volcano plot of RNA-seq(A) and RNC-seq (B). (C) Venn diagram of differentially expressed genes identified by RNA-seq and RNC-seq. (D) Network diagram of KEGG pathway enrichment associated with lipid metabolism and myoblast proliferation (Red indicates up-regulated RNC-mRNAs and blue indicates down-regulated RNC-mRNAs). (E) Number and type of encoded enzymes. **S3 Fig.** (A) The relationship between TR log2FC and mRNA log2FC. (B)Volcano map of TR. (C) Functional enrichment analysis of genes with very small TR differences (|FC | < 0.5). (D-E) Differences in the U, G, content of the 5’UTR of differentially expressed genes. (F) Molecular mechanism diagram. **S1 Table.** The number of genes and differentially expressed genes identified by RNA-seq and RNC-seq. **S2 Table.** Differential expression of translation initiation factors in two pig breeds.
(ZIP)

## Abbreviations

IMF: intramuscular fat
RNC-seq: ribosome nascent-chain complex-bound RNA sequencing
RNA-seq: RNA sequencing
TG: triglyceride
qRT-PCR: Quantitative real-time polymerase chain reaction
PVDF: polyvinylidene fluoride
ECL: Electrochemiluminescence
LM: Luchuan pig
DM: Duroc pig
