# Supplementary material for: Comparative analysis of translatomics and transcriptomics in the longissimus dorsi muscle of Luchuan and Duroc pigs
Source: PLoS One. 2025 Mar 18;20(3):e0319399. doi: 10.1371/journal.pone.0319399 (PMC11918432; doi:10.1371/journal.pone.0319399)
Supplement: S1 File — S1 Fig. (A) HeatMap of gene expression of each group. Using Pearson correlation coefficient, where red indicates r is close to -1 and blue indicates r is close to 1. (B) Gene expression density curve for each group. Log-transformation of RNA-seq and RNC-seq gene expression levels (rpkM) for each sample to reduce their dispersion, followed by the creation of density plots for each. (C, D) The linear relationship between mRNA abundance and RNC-mRNA abundance. (E) Principal Component Analysis (PCA) result. S2 Fig. (A, B) Volcano plot of RNA-seq(A) and RNC-seq (B). (C) Venn diagram of differentially expressed genes identified by RNA-seq and RNC-seq. (D) Network diagram of KEGG pathway enrichment associated with lipid metabolism and myoblast proliferation (Red indicates up-regulated RNC-mRNAs and blue indicates down-regulated RNC-mRNAs). (E) Number and type of encoded enzymes. S3 Fig. (A) The relationship between TR log2FC and mRNA log2FC. (B)Volcano map of TR. (C) Functional enrichment analysis of genes with very small TR differences (|FC | < 0.5). (D-E) Differences in the U, G, content of the 5’UTR of differentially expressed genes. (F) Molecular mechanism diagram. S1 Table. The number of genes and differentially expressed genes identified by RNA-seq and RNC-seq. S2 Table. Differential expression of translation initiation factors in two pig breeds. (ZIP) [file pone.0319399.s001.zip › Supporting Information/S1_fig.pdf.pdf]

S1 Fig.

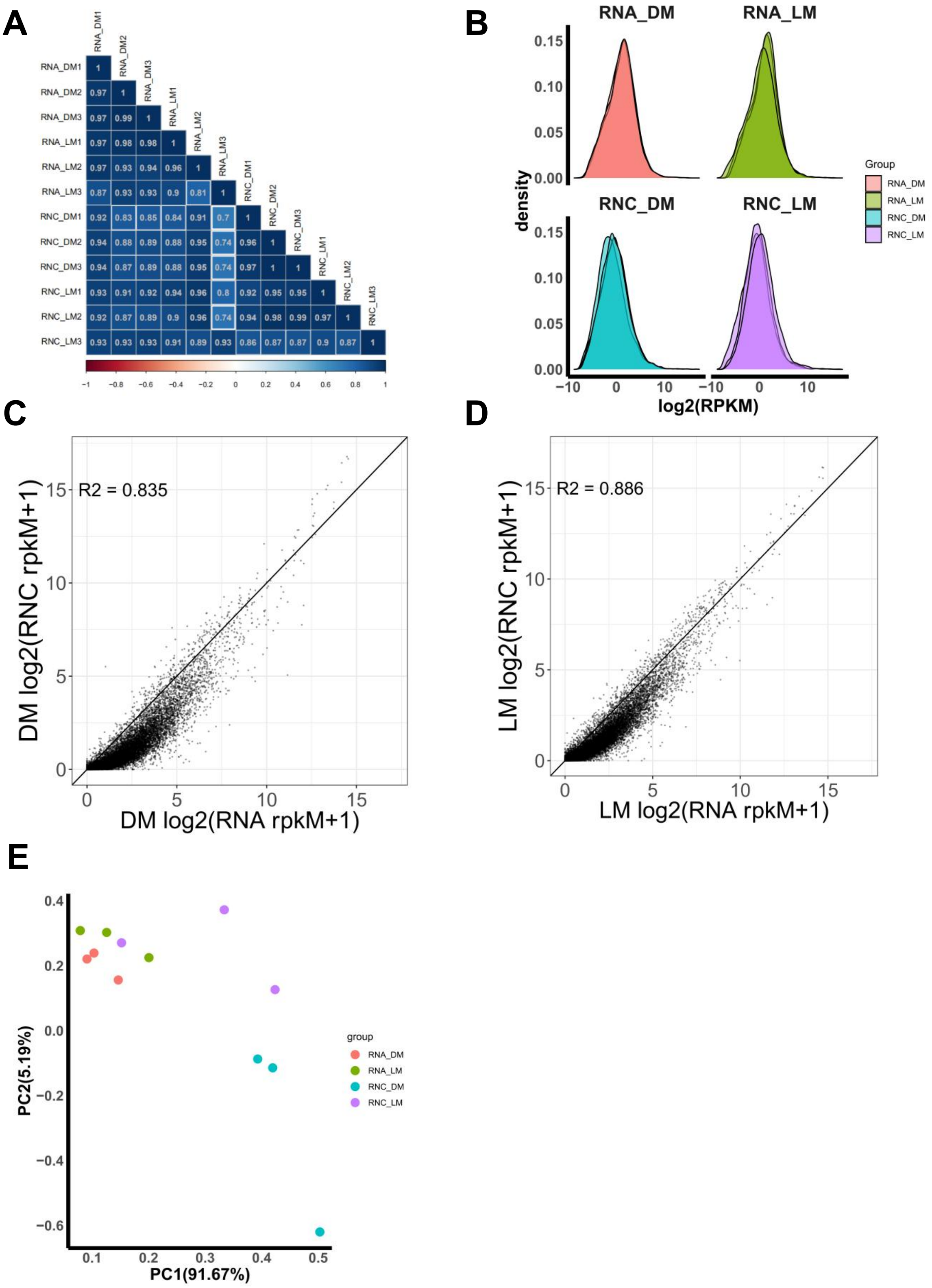

S1 Fig. (A) HeatMap of gene expression of each group. Using Pearson correlation coefficient, where red indicates r is close to -1 and blue indicates r is close to 1. (B) Gene expression density curve for each group. Log-transformation of RNA-seq and RNC-seq gene expression levels (rpkM) for each sample to reduce their dispersion, followed by the creation of density plots for each. (C, D) The linear relationship between mRNA abundance and RNC-mRNA abundance. (E) Principal Component Analysis (PCA) result.
